# Highly Selective and Efficient Transport of Au(III), Pt(IV), and Pd(II) from Hydrochloric Acid Across Polymer Inclusion Membranes Containing Ionic Liquid as Ion Carrier

**DOI:** 10.3390/membranes16030092

**Published:** 2026-03-02

**Authors:** Iwona Zawierucha, Cezary Kozlowski, Bernadeta Gajda, Katarzyna Witt

**Affiliations:** 1 Institute of Chemistry, Jan Dlugosz University in Czestochowa, 42-200 Czestochowa, Poland; c.kozlowski@ujd.edu.pl; 2 Department of Metallurgy and Materials Engineering, Czestochowa University of Technology, 42-200 Czestochowa, Poland; bernadeta.gajda@pcz.pl; 3 Faculty of Chemical Technology and Engineering, Bydgoszcz University of Science and Technology, 85-796 Bydgoszcz, Poland

**Keywords:** polymer inclusion membrane, ionic liquid, separation, Au(III) ions, Pd(II) ions, Pt(IV) ions

## Abstract

Ionic liquid (IL) N-methyl-N′-1-(4-t-butylphenylphosphinyl)butylimidazolium bis(trifluoromethylsulphonyl) imide was used for the first time as an ion carrier in membrane systems to selectively transport Au(III), Pt(IV), and Pd(II) ions. Au(III), Pd(II), and Pt(IV) were transported from HCl solutions utilizing a polymer inclusion membrane (PIM) with cellulose triacetate as the support, *o*-nitrophenyl pentyl ether as the plasticizer, and ionic liquid as the mentioned ion carrier. The modifications of source and receiving aqueous phase compositions are examined. High selectivity for Au(III) using the ionic liquid in the membrane was achieved at elevated HCl concentrations (≥0.5 M). When a 0.010 M KI solution was used as the receiving phase and a membrane with the optimal composition was applied, the extraction of Au(III) ions reached a maximum recovery rate of 93%**.** Moreover, PIM studies showed that carrier molecules doped in the membrane creates complexes with the Au(III) ion with a molar ratio of 1:1. The extractability of Au(III) through PIMs exceeded that of other metal ions, with the selectivity of transported metal ions ranked as follows: Au(III) >> Pt(IV), Pd(II). The recovery factors for gold, platinum, and palladium ions after 6 h of transport were 94%, 8%, and 1%, respectively.

## 1. Introduction

Precious metals are categorized as critical raw materials (CRMs), vital for the advancement of innovative technologies known as high tech [1]. Securing the availability of these raw materials is a key task of the policies of many countries. CRMs are considered to be those that are of great economic importance to the EU and are a high supply risk. The latest list of critical raw materials was published by the European Commission in 2023. It includes industrial and construction minerals, iron and iron alloys, precious metals, rare earth metals and other non-ferrous metals, “bio” materials, and others [2].

The unique properties of precious metals, such as Au, Ag, Pt, Pd, Rh, Ir, and Ru, i.e., excellent electrical conductivity, corrosion resistance, and catalytic activity, are crucial in industries [3]. These metals find application across various domains, which contributes to their high demand. For example, the common fields of use of gold are electronics and electrics (connectors, switches, and circuit boards) [4], medicine (dental works like fillings, crowns, and implants) [5], optics and photonics (coatings in mirrors and lenses) [6], and data storage and telecommunications (hard drives, antennas) [7]. The European Commission emphasizes the need to recover and recycle materials containing these substances as important activities for ensuring the continuity of their supply [2].

Au, Pt, and Pd occur as accompanying metals in copper ores and become concentrated in the final stage of metallurgical processing as anode sludge [8]. Copper anode sludge (CAS) is a valuable residue generated during the electrolytic refining of copper. Although it contains toxic elements such as As, Sb, and Cd, CAS is also a source of various valuable metals, including Au, Ag, and platinum-group metals, Se, Te, Sn, Cu, Ni, and Pb [9]. Waste materials containing precious metals also include waste from jewelry, modern electronics, and medical applications. Electronic waste, such as printed circuit boards (PCBs), is particularly rich in precious metals, containing over 60 elements, including precious metals (such as Au, Ag, Pd, and Pt), base metals (such as Cu, Sn, Ni, Fe, Al, Zn, Pb, Ti, and Co), and rare earth or critical elements (such as Ga, Ge, In, and Ta) [10,11]. Their recovery is not only economically beneficial but also strategically important in the context of the energy transition, which is largely based on clean technologies that do not always take raw material constraints into account.

An interesting and important application issue is the selective separation of noble metals, especially gold, from platinum and palladium ions from chloride solutions. Researchers are looking for the various methods that will be the most effective to do so. For example, Chung and Tabata [12] extracted Au(III) from Pd(II) and Pt(IV) using a 2-propanol–water mixture with NaCl in the concentration range of 2.5–4.0 mol/dm^3^. Gold(III) in the aqueous phase was quantitatively extracted, while the extraction of Pd(II) and Pt(IV) was much lower.

Kargari et al. [13] utilized a methyl isobutyl ketone (MIBK) as a selective carrier for gold(III) extraction, liquid paraffin as the membrane phase, sodium sulfite with sodium hydroxide as an internal phase reagent, and LK-80 as a natural and biodegradable emulsifier. The extraction results showed that only the Au(III) ions were transported across the liquid membrane from a model solution containing Au(III), Pd(II), Pt(IV), Cu(II), and Fe(III), which are usually present in the gold-containing stocks, such as anode mud.

In turn, Herrala et al. [14] evaluated the impact of oxidants (Cu, Fe), passive base metals (Ni, Zn, and Al), and precious metals (Pd, Pt, and Ag) contained in hydrometallurgical process streams on gold recovery through electrochemically assisted aqueous reduction (EAR). The study was carried out with synthetic solutions simulating chloride leachates. The described EAR method improved the selective recovery of gold from chloride solutions containing several bases and precious metals. Moreover, it was shown that the generated Fe(II) could act as a reductant for Au(III). Impurities of Al, Ni, and Zn had only a minor effect on gold recovery, while the deposition of Ag, Pd, and Pt was strongly inhibited by chloride. Thanks to this, high selectivity for gold recovery was achieved.

Polymer inclusion membranes (PIMs) are a well-known, eco-friendly membrane technique used, among others, for the selective extraction of diverse metal ions from aqueous solutions [15]. PIMs most often consist of three components: polymer matrix, plasticizer, and carrier [16]. The most significant is a carrier, whose role is to increase the effectiveness of the transport of the substance across PIMs from the source to the stripping phase [17]. In recent years, synthetic polymers (poly(vinyl chloride, polyethylene) and toxic solvents (chloroform, tetrahydrofuran) used to obtain the membranes have been replaced with biodegradable polymers (polylactide) [18] and green solvents (water/ethanol solution) [19], respectively. Furthermore, the choice of carrier is also essential because it cannot leak from the membrane structure and cause environmental pollution [20]. Currently, compounds from a group of ionic liquids (ILs) are often being used in this role [21]. They can be designed in such a way to distinguish the feature, which is the increased selectivity as well as efficiency in separating metal ions relative to commercial carriers [22]. The utilization of chosen ionic liquids (ILs) is in line with the trend of green chemistry and environmental sustainability (e.g., non-toxic N-alkyl cholinium-based ionic liquids, which showed no cytotoxicity up to 500 mmol/dm^3^ [23], but in the literature, an opposite suggestion can also be found [24]). It is important to remember that the environmental and safety profile of ionic liquids can vary significantly, depending on their specific chemical structure. While some ionic liquids pose minimal environmental risk, others can have a more serious environmental impact [22]. Kuroda [25] mentioned the toxicity of [C8mim]^+^-based ionic liquids. The value of EC_50_ (commonly used indicator of toxicity) for [C8mim]Cl for rat leukemia IPC-81 cells is 0.102 mmol/dm^3^; in comparison, the EC_50_ of methanol is 1584 mmol/L. [C8mim]Cl may be considered highly toxic.

However, different ionic liquids have varying EC_50_ values; not all are as toxic as [C8mim]Cl. Their toxicity depends on the length of the alkyl chain of the cation. It has been proven that the EC_50_ value decreases exponentially as the alkyl chain length increases from 2 to 10 carbon atoms. Moreover, the addition of a substituent to the alkyl chain, which increases polarity, can reduce the toxicity of ionic liquid by an order of magnitude [25].

Imidazole is a heterocyclic compound with a five-membered planar ring that is amphoteric. Imidazoles can bind to metal ions; therefore, these substances are utilized as extractants in solvent extraction and as ion carriers in PIMs [26], e.g., for the separation and removal of various metal ions from aqueous solutions [27]. Imidazole compounds have recently been used to develop new reactive carriers by obtaining ion liquids. For example, Szczepański et al. [28] synthesized three ionic liquids based on bromide anion and imidazolium cation derivatives with different lengths of alkyl chains (C1, C4, and C8) and effectively used them to remove Cd(II) from a mixed water solution of four metal ions. The feed phase was composed of 0.002 M Cd(II), Cu(II), Pb(II), and Zn(II) chlorides dissolved in HCl (0.5 or 1 M solution). Authors demonstrated that RILC_8__Br ionic liquid showed the selective transport of Cd(II) ions and their active concentration in the stripping solution, with the fluxes reaching a value of 2.7 × 10^−10^ mol/cm^2^ s.

In turn, Zhou et al. [29] prepared imidazole ionic liquid-grafted cellulose nanocrystals (IL@CNC) and used them as a nanofiller in modifying composite membranes based on quaternized chitosan (QCS) and polyvinyl alcohol (PVA). The imidazolium salt-based ionic liquid was synthesized in a Menshutkin reaction from 1-methylimidazole and 3-chloropropyltriethoxysilane. Then, to the prepared IL, the CNC powder was added, and after washing in ethanol and drying, the IL@CNC was obtained. The researchers demonstrated that the ion exchange capacity was enhanced with the help of the 1D shape of CNC, together with the grafted imidazole groups, that highly effective ion transport channels inside the membrane. That increased ion transport across the membrane.

Alcalde et al. [30] incorporated imidazolium-polymerized ionic liquids (PILs) into a polymeric matrix. They used an original acrylate formulation to copolymerize a series of imidazolium-based PILs. PIL-1, PIL-2, and PIL-4 have the same anion, Tf_2_N^−^, but differ in cation due to differences in chain length (in the case of PIL-1 and PIL-2) or the presence of a triazole fragment (in the case of PIL-4). The PIL with the longest aliphatic chain produced dense, homogeneous membranes that were suitable for studies on toxic Cr(VI) ion recovery from industrial wastewater. This membrane showed high efficiency (from 63% to 82%) in recovering chromate from industrial wastewater samples, which highlights its practical applicability in environmental remediation.

Babu et al. [31] used a carboxyl-functionalized imidazolium-based ionic liquid, 1-carboxymethyl-3-dodecyl imidazolium bromide [C_12_C_1_COOHim]Br, for the selective recovery of gold (Au) and copper (Cu) from discarded computer pins. The dual-function ionic liquid acted as a sorbent for gold through anion exchange and as a leaching agent for copper via coordination with carboxylic acid groups. Gold with a purity of 97% was efficiently recovered from aqua regia-leached solutions at room temperature, with a rate of 96.7%. Furthermore, copper was selectively leached from an aqueous medium at 65 °C, with a recovery rate of 99.1%.

This article describes the results of using, for the first time, the synthesized ionic liquid—an imidazole, tertiary phosphine oxide with a terminal amino group, i.e., N-methyl-N′-1-(4-t-butylphenylphosphinyl)butylimidazolium bis(trifluoromethylsulphonyl) imide (IL), as the ion carrier in a PIM system for the selective transport of Au(III), Pt(IV), and Pd(II). The primary objective of this work was to develop and optimize a PIM system with the IL as the carrier for the effective extraction and transport of Au(III) ions. In addition to characterizing the extraction behavior, particular emphasis was placed on optimizing membrane composition and operational parameters in order to enhance transport efficiency. Furthermore, the selectivity of the proposed system toward Au(III) in the presence of accompanying metal ions was also evaluated.

## 2. Materials and Methods

### 2.1. Reagents

**Figure 1:**
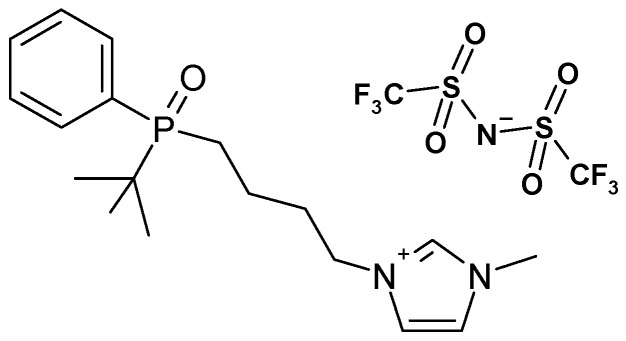
Structure of investigated ionic liquid.

Inorganic chemicals, i.e., gold chloride (AuCl_3_), palladium chloride (PdCl_2_), platinum chloride (PtCl_4_), hydrochloric acid (HCl), and potassium iodide (KI) of analytical grade were purchased from POCh (Gliwice, Poland). Analytical-grade organic chemicals, such as dichloromethane (DCM), cellulose triacetate (CTA), and *o*-nitrophenyl pentyl ether (*o*-NPPE), were acquired from Fluka, Seelze, Germany, and applied without further processing. Double distillation water was used to create aqueous solutions with a 0.1 µS/cm conductivity. The carrier (Figure 1), a tertiary phosphine oxide with a terminal amino group, was obtained and patented by Professor Drabowicz’s team (patent pending P-411260).

### 2.2. Preparation of Polymer Inclusion Membranes

The methodology for the preparation of polymer inclusion membranes was executed in accordance with the procedures delineated in prior studies [32,33]. PIMs were prepared using CTA in DCM (0.625 g in 50 cm^3^)—matrix, 10% *v*/*v* solution of *o*-NPPE in DCM as a plasticizer, and 0.05 M solution of IL in DCM as a carrier. The specific portions of CTA, plasticizer, and carrier solutions were mixed and poured into a 5.0 cm glass ring, which was secured to a glass plate using a CTA–dichloromethane adhesive. To allow DCM to evaporate at room temperature, the glass ring was permitted to rest overnight. By soaking it in cold water, the resulting membrane was separated from the glass plate. The membrane’s effective surface area was 4.9 cm^2^. The carrier concentration in the membrane varied in the range of 0.01–0.75 M, corresponding to a carrier content in the PIM of 3–10 wt%. The membrane thickness was determined using an Inco-Veritas A2002M-type digital ultrameter, with a standard variation of 1.0 μm. The average thickness of the CTA membrane was 24 ± 0.9 μm. Prior to experiments, membranes were conditioned in demineralized water for 24 h.

### 2.3. Membrane Characterization

The FTIR-ATR spectra of the membrane with optimal composition were recorded using a Bruker Alpha-PFT-IR device with a diamond attenuated total reflectance (ATR) accessory and were recorded within a wavenumber range of 500–4000 cm^−1^. The membrane surface morphology at the nanometer scale was analyzed using a NanoScope IIIa atomic force microscope (AFM) (Veeco, Plainview, NY, USA) operating in tapping mode and equipped with an SPM scanning probe (Veeco, Digital Instruments USA, Plainview, NY, USA). Image processing was performed using AFM NanoScope v.7.20 (Digital Instruments Veeco Metrology Group, New York, NY, USA) software.

### 2.4. Transport Studies

Transport experiments were conducted in a permeation module cell, as described in our previous work [16]. The membrane film was securely clamped between the two compartments of the cell. Both the feed and receiving aqueous phases (50 cm^3^ each) were continuously stirred at 600 rpm. The aqueous receiving phase was a KI solution, whereas the source phase was an aqueous solution of Au(III) ions in hydrochloric acid medium. The transport was carried out at room temperature (23–25 °C). Using a syringe and a sampling port, samples of the aqueous phases were regularly taken out and examined to ascertain the concentration of metal ions.

Kinetics of the transport process through PIMs are described by a first-order reaction with respect to the metal ion concentration [34]:

(1) $$\ln\left(\frac{c}{c_{0}}\right)=-kt$$

where *c* is the metal ion concentration (mol/dm^3^) in the source phase at a specific time, *c*_0_ is the initial metal concentration in the source phase (mol/dm^3^), *k* is the rate constant (s^−1^), and *t* is the transport time (s).

A graph of *ln*(*c*/*c*_0_) versus time was created in order to get the *k* value. The following formula was used to determine the permeability coefficient (*P*) [35]:

(2) $$P=-\frac{V}{A}k$$

where *V* is the volume of aqueous source phase (m^3^), and *A* is the surface area of the membrane (m^2^). The initial flux (*J*_0_) in (µmol/m^2^ s) was determined to be equal to [36]:

(3) $$J_{0}=Pc_{0}$$

The recovery factor (*RF*), which characterizes the effectiveness of metal ion removal from the source phase, was computed as follows:

(4) $$RF=\frac{c_{0}-c}{c_{0}}\times 100\%$$

The selectivity parameters *S_MI_*/*_MII_*, which are determined by Equation (5), specify the extraction of metal ions via the PIM (where *J*_0,*MI*_ and *J*_0,*MII*_ are the initial flux for the MI and MII metal ions, respectively).

(5) $$S_{M_{I}/M_{II}}=\frac{J_{0,M_{I}}}{J_{0,M_{II}}}$$

The flame atomic absorption spectrometer (Solar 939, Unicam, Munich, Germany) was used to determine metal ion concentrations. The observed mean variance was less than 2%, and the values provided match the average of the triplicates.

## 3. Results and Discussion

### 3.1. Membrane Characterization

**Figure 2:**
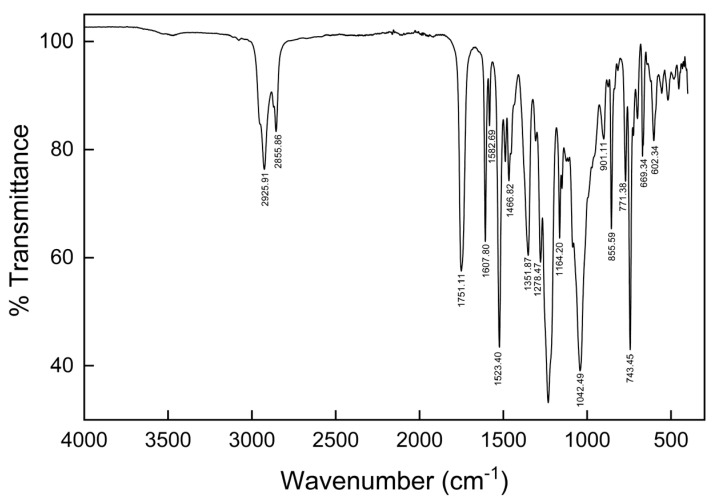
The infrared spectra of tested membrane.

The peaks presented in the FTIR-ATR spectrum (Figure 2) are related to characteristic bonds that exist in individual components in the membrane: a polymer—CTA; a plasticizer—*o*-NPPE; and the carrier—N-methyl-N′-1-(4-t-butylphenylphosphinyl)butylimidazolium bis(trifluoromethylsulphonyl) imide.

The FTIR spectrum for the tested membrane is characterized by the overlap of bands of all components. The presence of characteristic bands, such as RC-CH (602.34 cm^−1^ and 669.93 cm^−1^), C-O stretch (1278.47 cm^−1^), C=O stretch (1751.11 cm^−1^), -CH_2_-CH_3_- stretch (2855.86 cm^−1^), and -CH_2_- (2925.91 cm^−1^), confirms that the membrane was made from cellulose triacetate. The presence of the R_2_C=CH_2_ (885.59 cm^−1^), -CH_2_- alkanes bands (1466.82 cm^−1^), N-O aromatic nitro group (1523.40 cm^−1^), N=O nitroso group (1582.69 cm^−1^), and C=C stretch bands (1607.80 cm^−1^) confirms the use of the 2-nitrophenyl pentyl ether as a plasticizer. The carrier shows, in turn, peaks related to P-OR (901.11 cm^−1^), C-F (1040.20 cm^−1^), C=S (1164.24 cm^−1^), P=O (1240.02 cm^−1^), and S=O (1351.87 cm^−1^) bands. The obtained data are consistent with the literature [37,38,39], which describes cellulose triacetate membranes with nitrophenyl plasticizers. The authors mentioned a strong stretching vibration band of carbonyl (C=O) groups originating from the acetate groups (in the region 1740–1755 cm^−1^), the asymmetric stretching vibrations of C-O-C ester bonds (1220–1237 cm^−1^), and stretching vibrations of methyl C-H groups (2890–2960 cm^−1^) derived from CTA molecules. The presence of plasticizers (NPPE or NPOE) is confirmed by the existence of a strong asymmetric stretching vibration band (~1520 cm^−1^) of the nitro group (-NO_2_), skeletal stretching vibrations of the C=C bonds of the aromatic ring (1600–1615 cm^−1^), and out-of-plane bending vibrations of the C-H bonds in the aromatic ring (~850 cm^−1^).

**Figure 3:**
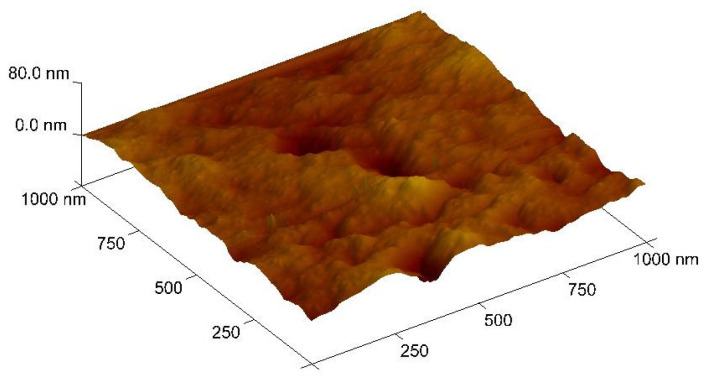
Image of atomic force microscopy (AFM) for the CTA/ONPPE/IL membrane.

An AFM image of the surface of the CTA/ONPPE/IL membrane is presented in Figure 3.

For this PIM, a homogenous saturation of IL was found, limiting potential heterogeneous behavior of the inclusion organic phase into the membrane matrix, a consequence of the slow DCM evaporation process during the PIM preparation, leading to the formation of very small pores that were less than few μm and were thus invisible on AFM acquisitions.

### 3.2. Transport of Au(III) from Hydrochloric Acid Solution by PIM

Studies were carried out on the transport of Au(III) ions from aqueous chloride solutions through PIMs using ionic liquid (IL). The main objective of the study was to examine the kinetics and efficiency of the transport process for Au(III) ions through polymer inclusion membranes (PIMs). Additionally, the study aimed to assess how the composition of the source and receiving phases, as well as the content of the carrier in the membrane, affects the efficiency of the Au(III) ion separation process from aqueous hydrochloric acid solutions.

#### 3.2.1. Modification of the Source Aqueous Phase Composition

**Figure 4:**
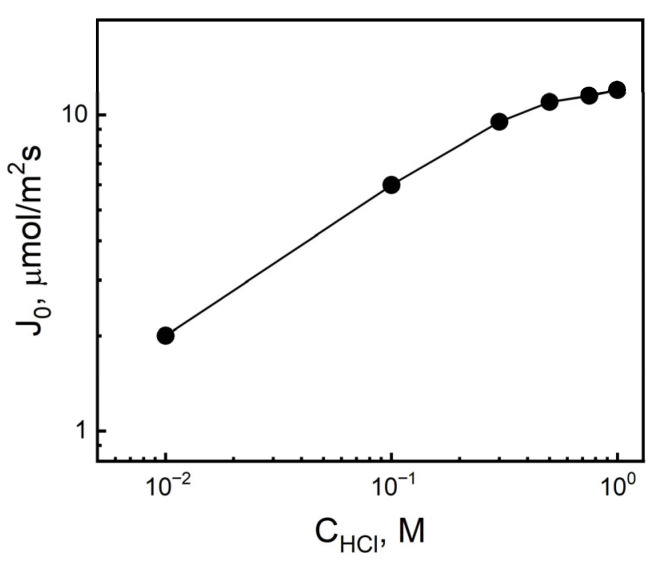
Dependence of the Au(III) initial fluxes in transport through PIM on the HCl concentration in the source phase. Source phase: 1.0 × 10^−4^ M Au(III) solution; PIM: 2.0 cm^3^ *o*-NPPE/1.0 g CTA; and 0.25 M IL. Receiving phase: 0.01 M KI. PIM composition (wt%): 59% plasticizer, 36% matrix, and 5% carrier.

Studies were conducted on the influence of the composition of the source phase, i.e., HCl and Au(III) concentrations, on the transport parameters of gold through polymer inclusion membranes containing IL. First, the influence of the HCl concentration in the source phase in the range of 0.1 ÷ 1.0 M HCl on the transport of Au(III) ions through PIMs was examined. Figure 4 shows the dependence of the initial values of Au(III) ion transfer fluxes on the HCl concentration in the source phase using a 1.0 × 10^−4^ M Au(III) solution as the source phase and a 0.01 M KI solution as the receiving phase.

The logarithm values of the initial fluxes of Au(III) ion transport increase with increasing Cl^−^ concentration in the range of 10^−2^ to 0.5 M. The maximum initial flux of Au(III) ion transport from the aqueous source phase containing 1 × 10^−4^ M of these ions through the PIM was 11.2 µmol/m^2^∙s for 0.5 M HCl. In the concentration range of 0.5 to 1.0 M Cl^−^, the Au(III) ion transport flux stabilized its value. Above the Cl^−^ ion concentration of 0.5 M, the share of IL anion exchange with Cl^−^ ions of the carrier molecule was smaller, resulting in minor changes in the values (Ji) for measurements above 0.5 M Cl^−^. This demonstrates a common transport mechanism based on the formation of an IL ion pair with ${\mathrm{AuCl}}_{4}^{-}$ ions. The change in rate with the concentration (acidity) for IL is therefore typical for the co-transport and neutralization of complex molecules in the membrane: Cl^−^ − IL^+^ + ${\mathrm{AuCl}}_{4}^{-}$ = (IL^+^ − ${\mathrm{AuCl}}_{4}^{-}$)_mem_ + Cl^−^, where IL denotes an ionic liquid molecule. The effect of the initial Au(III) concentration in the source phase on the rate of transfer of these metal ions by the PIM was also investigated. For this purpose, four gold solutions were prepared in a 0.1 M HCl solution containing 1.0 × 10^−4^ M ÷ 1.0 × 10^−3^ M Au(III). The receiving phase was a 0.010 M KI solution.

The transport kinetics parameters, i.e., rate constant (k) and initial flux (Ji), are presented in Table 1.

**Figure 5:**
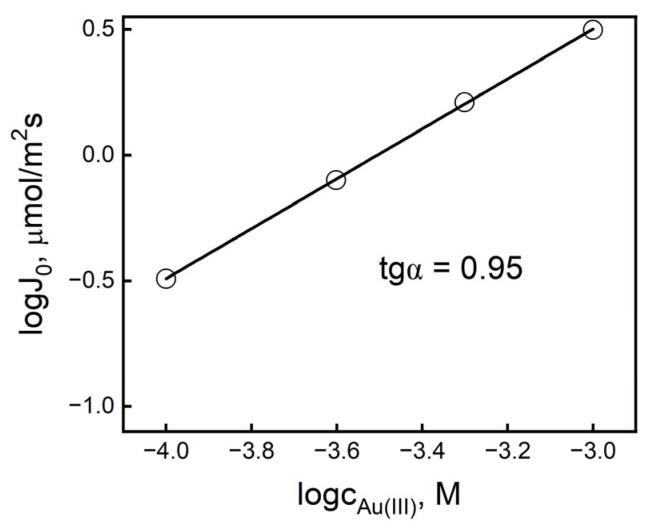
Relation of the initial Au(III) ion flux value on the metal concentration in the source phase (log–log) in transport through PIM with IL; process conditions the same as in Table 1.

Figure 5 shows the obtained linear dependences of the J_0_ value on the Au(III) concentration in the log–log system, consistent with the transport conditions described in Table 1.

The changes in the values of the initial fluxes of Au(III) ion transport through the PIM as a function of the metal concentration in the source phase (c_0_) in the logarithmic system are linear, with the slope coefficient corresponding to the stoichiometric coefficient for gold(III) ions in the complexation reaction equation in the membrane phase. The tangent of the angle of inclination of the straight line determined for IL was 0.95. The obtained values of the angles of inclination of the straight line presented in Figure 5 indicate that one ${\mathrm{AuCl}}_{4}^{-}$ ion participates in the reactions occurring at the interface of the source phase and the membrane; thus, this reaction is described by a first-order equation due to the metal concentration.

#### 3.2.2. Receiving Aqueous Phase Modification

Iodide ions (often from potassium iodide) react with gold to form a soluble gold–iodide complex, typically [AuI_4_]^−^. This allows the gold to be back-extracted from the membrane.

**Figure 6:**
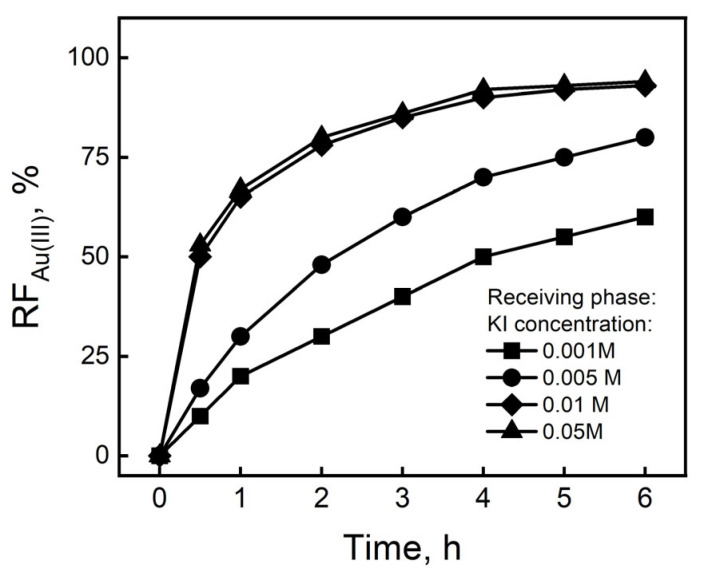
Relation of recovery factors (*RF*) vs. time transport of Au(III) across PIMs containing IL. Source phase: 5.0 × 10^−4^ M Au(III) in 0.5 M HCl. Membrane: 2.0 cm^3^ *o*-NPPE/1.0 g CTA; 0.25 M IL. Receiving phase: solutions of KI. PIM composition (wt%): 59% plasticizer, 36% matrix, and 5% carrier.

The process of Au(III) ion transport through polymer inclusion membranes containing IL was investigated using a 0.01 M KI solution as the receiving phase. As can be seen from the data presented in Figure 6, after 6 h of the process, Au(III) ions were released at 89% when 0.01 and 0.05 M KI solutions were used as the receiving phase. However, when using solutions with lower KI concentrations, the degree of Au(III) ion removal was much lower and remained below 76%.

### 3.3. Effect of Carrier Concentration on Au(III) Ion Transport Efficiency

**Figure 7:**
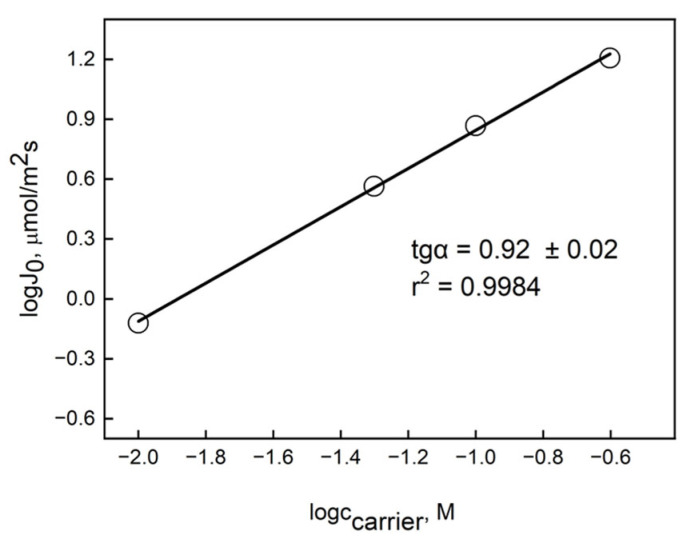
Logarithmic dependence of the initial flux values on the IL carrier concentration in the membrane; source phase: 2.0 × 10^−4^ M Au(III) in 0.5 M HCl; membrane: 2.0 cm^3^ *o*-NPPE/1.0 g CTA; and receiving phase: 0.01 M KI.

The effect of the IL carrier concentration in PIMs on the permeation of Au(III) ions was examined. Consistent with prior research, the source phase comprised a 5.0 × 10^−4^ M Au(III) solution in 0.5 M HCl, while the receiving phase consisted of a 0.01 M KI solution. Membranes were prepared with a fixed content of CTA (20 mg) and plasticizer (2.0 cm^3^ *o*-NPPE per 1.0 g CTA), while the concentration of the carrier in the membrane ranged from 0.01 M to 0.75 M. The transport studies were conducted over a duration of six hours. Membranes lacking a carrier did not facilitate the transport of Au(III) ions, suggesting that the concentration of Au(III) within the membrane influences the facilitated transport of metal ions by the PIM. Table 2 presents the values of transport kinetic parameters, including the rate constant (*k*) and initial flux (*J_0_*) of Au(III) ions through PIMs containing IL. Figure 7 illustrates the relationship between the transport flux of Au(III) ions through the polymer inclusion membrane and the concentration of the carrier within the membrane. The data in Table 2 indicate that an increase in carrier concentration within the membrane correlates with a rise in the initial flux of Au(III) transfer through the membrane. A carrier concentration of 0.75 M in the membrane, determined based on the total volume of plasticizer and carrier, results in the saturation of the polymer inclusion membrane with the carrier. The concentration of the carrier in the membrane yielded the highest initial flux values for Au(III) ion transfer, measured at 18.80 µmol/m^2^∙s. The membrane with an optimal composition of 56% plasticizer, 34% polymer matrix, and 10% carrier (wt%) exhibited the fastest transport rate compared to a PVC-based PIM containing Cyphos^®^ IL 104 [40].

Figure 7 shows the dependence of the initial flux value (J_0_) on the concentration of carrier IL in the membrane in a log–log system, within the range of carrier concentrations from 0.01 to 0.75 M. Determining the stoichiometry of the transported complex allows for establishing the dependence of the logarithm of the initial flux value (J_0_) on the logarithm of the carrier concentration for the Au(III) complex with IL. The parameter “a” was determined from a linear regression based on the tangent of the slope angle α, indicating the number of carrier molecules combined with the metal ion in the membrane phase. As can be seen from the data presented in Figure 7, the determined values of “a” from the regression equations were obtained with high determination coefficients (r^2^), and they also have low standard deviation errors, which demonstrates a high level of statistical significance for the correlation.

The elevated concentration of the IL carrier in the PIM led to a decrease in the parameters affecting the transport rate of Au(III) ions. A documented case in the literature indicates that the increased viscosity of the organic membrane phase elevates membrane resistance, thereby restricting the diffusion of the complex through the membrane [41]. A shift in the transport mechanism from diffusive to stepwise is observed, attributed to the crystallization of the carrier within the membrane [42]. The logarithmic relationship between the initial flux value and the carrier concentration in the membrane enabled the determination of the stoichiometry of the complexes formed during metal ion transport through the membrane. The tangent angle α (slope) for IL suggests that the complex in the organic phase has a composition of M:IL = 1:1.

### 3.4. Competitive Separation of Pt(IV), Pd(II), and Au(III) Ions

**Figure 8:**
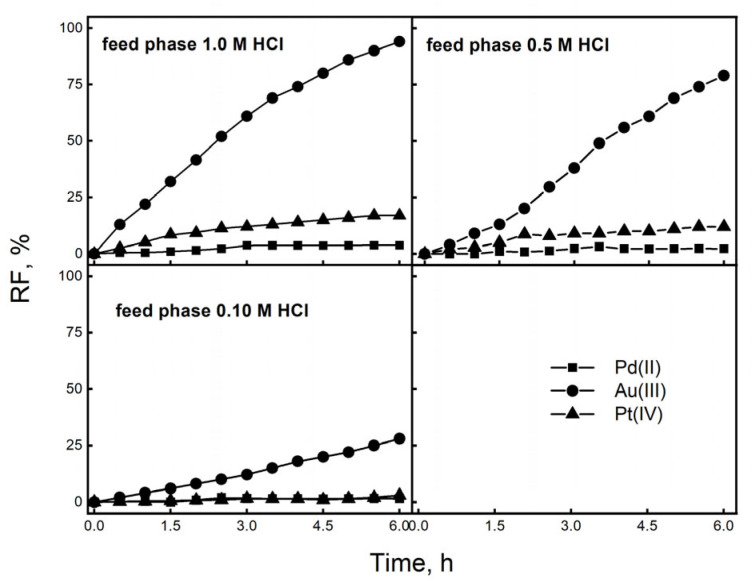
RF values determined from competitive transport of Au(III), Pt(IV), and Pd(II) ions through PIM. Source phase: 5.0 × 10^−4^ M solution of Au(III), Pd(II), and Pt(IV); membrane phase: 2.0 cm^3^ *o*-NPPE/1.0 g CTA, 0.5 M IL (carrier); and receiving phase: 0.01 M KI. PIM composition (wt%): 57% plasticizer, 34% matrix, and 9% carrier.

The next stage of the study was to determine the separation capacity of IL in the transport of metal ions from the source phase containing a 5.0 × 10^−4^ M solution of Au(III), Pt(IV), and Pd(II) chlorides in solutions of 0.1, 0.5, and 1.0 M HCl. For this purpose, a PIM containing a 0.5 M IL carrier and 2.0 cm^3^ of *o*-NPPE/1.0 g of CTA was used. The receiving phase was 0.01 M KI. Figure 8 presents the values of the recovery factor (RF) of metal ions during the competitive transport of ions from the solution containing Au(III), Pt(IV), and Pd(II) as a function of time.

The recovery rates for gold(III) ions are much higher than those for platinum(IV) and palladium(II) ions, with separation from solutions of 0.1, 0.5, and 1.0 M HCl resulting in recovery rates of 6%, 24%, and 93%, respectively. The initial flux of Au(III) ions during transport through the PIM containing IL reached maximum values of 15.8 µmol/m^2^·s, indicating a highly efficient transport mechanism. This efficiency indicates that the PIM with ionic liquid might be a good option for selectively recovering gold(III) ions in industrial uses. Conversely, the transport of Pt(IV) from 1.0 M HCl through the PIM containing IL yielded an RF value of 13%. The results from moving ions through PIMs from mixtures show that using IL greatly improves the ability to separate gold(III) ions, with separation factors going over 100 (Table 3).

Table 3 presents the selectivity series and separation coefficients determined based on the initial transport flux values according to the conditions presented in Figure 6. The data shows that Au(III) ions are effectively separated when moving through the membrane with IL, but for the other metals, the initial ion transfer rates were all below 1.8 µmol/m^2^·s. The initial transport flux values for Au(III) through membranes with an IL carrier from HCl solutions at concentrations of 0.1, 0.5, and 1.0 M were 2.9, 6.2, and 15.8 µmol/m^2^·s, respectively.

High values of separation coefficients of Au(III) compared to other metals show that obtained membranes are very selective, with a preference order of Au(III) > Pt(IV) > Pd(II), which is linked to how strongly IL molecules attract the anionic form of gold(III) ions. During transport using IL, we observed a high selectivity of gold(III) ions for palladium(II), along with a simultaneous high Au(III) transfer rate, effectively separating these metals from a 1.0 M HCl solution (S_Au(III)/Pd(II)_) = 122).

A recovery and separation study of gold, palladium, and platinum halogenated complexes using a tetraionic liquid carrier demonstrated excellent extractability of gold via an anion exchange mechanism. The results of the present study indicate that the gold complex is particularly suitable for the efficient and selective separation of gold from palladium and platinum in mixed solutions, representing a finding that differs from observations reported by Boudesocque et al. (2019) [43].

## 4. Conclusions

This research examined the use of an immobilized membrane (PIM) with ionic liquid (IL) as an ion carrier for the transport of Au(III) from the source phase, which consists of hydrochloric acid solutions. The highest transport rate (18.80 µmol/m^2^∙s) and the greatest Au(III) removal (96%) were achieved using a membrane with the optimal composition (wt%), 56% plasticizer, 34% polymer matrix, and 10% carrier, at an HCl concentration of 0.5 M in the source solution. The chemical composition of a complex transported via the membrane was determined, in which the ratio between the IL molecule and Au(III) ion was 1:1. The best concentration of the stripping agent in the receiving phase was identified, with a solution of 0.010 M KI producing the maximum initial flux. As a result of the reaction between Au(III) and I^−^ in the receiving phase, a complex [AuI_4_]^−^ is formed, accelerating the transfer of gold from the membrane. The limited interaction of IL molecules with Pt(IV) and Pd(II) ions is associated with the elevated separation coefficients of Au(III) in comparison to the other metal ions examined. The series Au(III) > Pt(IV) > Pd(II) defines the transport rate of the measured ions, particularly when the separation coefficients are more than 100.

## Figures and Tables

**Table 1:** Values of the rate constants and initial fluxes of Au(III) ion transfer through PIMs containing IL depend on the initial metal ion concentration in the source phase. Membrane phase: 2.0 cm^3^ *o*-NPPE/1.0 g CTA; 0.25 M IL. Receiving phase: 0.01 M KI. PIM composition (wt%): 59% plasticizer, 36% matrix, and 5% carrier.

Au(III) [M]	Rate Constants, k [h^−1^]	Initial Fluxes, J_0_ [µmol/m^2^∙s]
1.0 × 10^−4^	5.81 × 10^−6^	0.098
2.5 × 10^−4^	1.42 × 10^−5^	0.617
5.0 × 10^−4^	2.90 × 10^−5^	2.630
1.0 × 10^−3^	5.68 × 10^−5^	9.550

**Table 2:** Values of the rate constants and initial Au(III) ion transfer fluxes through PIMs containing IL depend on the carrier concentration in the membrane.

IL Concen. in PIM, [M]	Rate Constants, *k* [h^−1^]	Initial Fluxes, *J*_0_ [µmol/m^2^∙s]
0.01	4.72 × 10^−7^	1.65
0.05	2.86 × 10^−6^	4.36
0.10	5.27 × 10^−6^	6.99
0.25	1.27 × 10^−5^	16.12
0.50	1.52 × 10^−5^	18.40
0.75	1.60 × 10^−5^	18.80

**Table 3:** Selectivity orders and separation coefficients (S) determined based on the initial flux values (J_0_) during the transport of Au(III), Pt(IV), and Pd(II) through PIM.

Concn. of HCl in Source Phase	Initial Fluxes, J_0_, [µmol/m^2^∙s]	Selectivity Order and Coefficients
0.1 M	JAu(III) = 2.90 JPt(IV) = 0.13 JPd(II) = 0.02	$\begin{array}{ccc} & 22 & \\ Au\left(III\right) & > & Pt\left(IV\right) \end{array}\begin{array}{cc} 145 & \\ > & Pd(II) \end{array}$
0.5 M	JAu(III) = 6.21 JPt(IV) = 0.20 JPd(II) = 0.10	$\begin{array}{ccc} & 31 & \\ Au\left(III\right) & > & Pt\left(IV\right) \end{array}\begin{array}{cc} 62 & \\ > & Pd(II) \end{array}$
1.0 M	JAu(III) = 15.8 JPt(IV) = 1.80 JPd(II) = 0.13	$\begin{array}{ccc} & 9 & \\ Au\left(III\right) & > & Pt\left(IV\right) \end{array}\begin{array}{cc} 122 & \\ > & Pd(II) \end{array}$

## Data Availability

The original contributions presented in the study are included in the article, further inquiries can be directed to the corresponding author.
